# Cardiovascular Risk Associated with Menopause and Menopause Hormone Therapy: A Review and Contemporary Approach to Risk Assessment

**DOI:** 10.1007/s11883-025-01343-6

**Published:** 2025-10-09

**Authors:** Zoee D’Costa, Emily Spertus, Shipra Hingorany, Rajita Patil, Tamara Horwich, Marcella Calfon Press, Janki Shah, Karol E. Watson, Lua Jafari

**Affiliations:** 1 David Geffen School of Medicine, Department of Medicine, University of California, Los Angeles, Los Angeles, CA USA; 2 University of California, Berkeley, Berkeley, CA USA; 3 Department of Medicine, Division of Cardiology, University of California, Los Angeles, Los Angeles, CA USA; 4 Department of Obstetrics and Gynecology, University of California, Los Angeles, Los Angeles, CA USA

**Keywords:** Menopause, Hormone, Cardiovascular risk, Menopausal hormone therapy (MHT), ASCVD, Algorithm

## Abstract

**Purpose of Review:**

Discuss the effects of menopause and menopause hormone therapy (MHT) on cardiovascular risk, and propose a structured, person-centered framework for cardiovascular risk assessment when initiating MHT.

**Recent Findings:**

The risk of atherosclerotic heart disease accelerates during the menopause transition due to hormonal, metabolic, and vascular changes. Both menopause and MHT affect cardiovascular risk factors (i.e. blood pressure, lipids, insulin resistance) and cardiovascular events (i.e. myocardial infarction and stroke). Early clinical trial evidence demonstrated that oral synthetic MHT, including conjugated equine estrogen (CEE) with medroxyprogesterone acetate (MPA), is associated with increased coronary heart disease and stroke risk, particularly in older, postmenopausal women. Contemporary formulations such as low-dose transdermal estrogen and micronized progesterone have lower cardiovascular risk. A personalized assessment when initiating MHT should consider age, time since menopause, baseline cardiovascular (CV) risk, and choice of MHT formulation. Assessment of baseline CV risk should include a comprehensive review of traditional CV risk factors and consideration of risk-enhancing factors (including female-specific risk factors) and imaging for subclinical atherosclerosis (i.e. coronary artery calcium scoring) to provide a person-centered risk assessment.

**Summary:**

Menopause is an important period to implement prevention strategies to reduce future incidence CVD. A structured, individualized approach that accounts for the timing, formulation and delivery of MHT can optimize cardiovascular safety. This review provides a framework for personalized decision-making and highlights the need for further research to clarify MHT’s impact on long-term CV outcomes.

**Graphical Abstract:**

**Figure:**
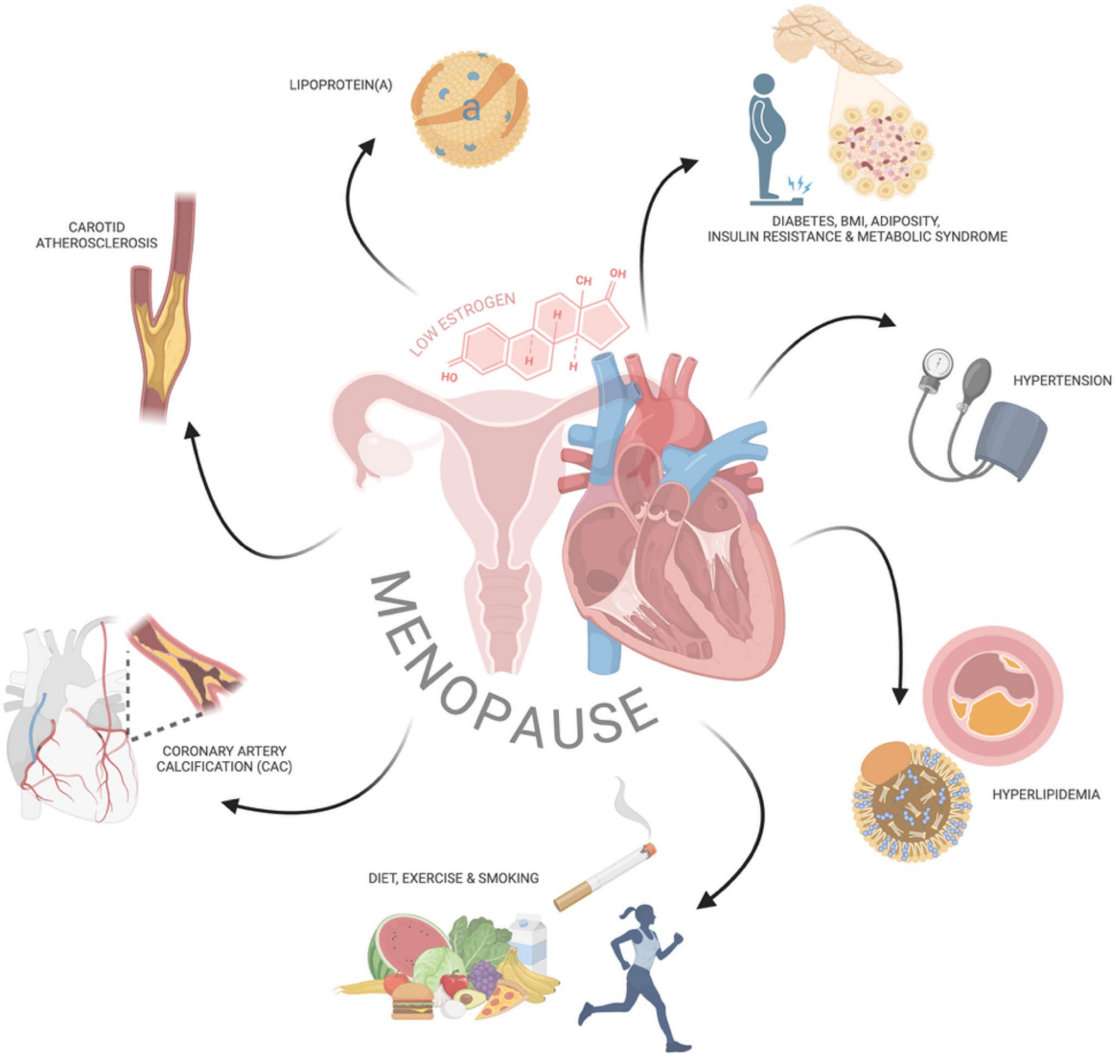
Visual Model of Cardiovascular Risk Factors Influencing Menopausal Hormone Therapy Decisions. This conceptual figure displays the intersection of cardiovascular and reproductive health during menopause. Radiating outward are key cardiovascular risk factors influenced by both menopause and menopausalhormone therapy (MHT), including blood pressure, lipid profile changes, insulin resistance, adiposity, coronary artery calcium (CAC), carotid atherosclerosis, Lp(a), and lifestyle behaviors. This visually integrated model highlights the need for individualized, risk-stratified assessment when considering MHT, with attention to as many of these factors as possible. Created in BioRender. D’Costa, Z. (2025)

## Introduction

Cardiovascular disease (CVD) is the leading cause of death in women [1, 2]. Many women develop coronary artery disease (CAD) later than men, typically during midlife, with 60% of women nationally seeking care for cardiac conditions at this stage [2, 3]. Menopause represents a pivotal physiologic transition, commonly accompanied by symptoms that prompt a significant number of people to seek clinical care. These encounters offer a critical window for comprehensive cardiovascular risk assessment and preventive interventions to mitigate long-term risk. Menopause induces a constellation of hormonal, psychosocial, and physiologic changes – including vasomotor symptoms, sleep disturbances, mood changes, and metabolic shifts – that can negatively impact quality of life and accelerate risk for future CVD [4, 5]. Worsening cardiovascular (CV) risk factors inherent to the menopause transition promote vascular vulnerability and likely accelerate atherosclerosis [4]. Therefore, the menopause transition is an important window of opportunity for implementing strategies to prevent cardiovascular disease.

Historically, menopause hormone therapy (MHT) has been used to alleviate menopause symptoms, though its oral synthetic formulations were associated with increased CV events, including coronary heart disease (CHD) and stroke, in older postmenopausal women [6]. Contemporary data on transdermal estrogen and micronized progesterone MHT formulations have been shown to have lower cardiovascular risks than oral and synthetic formulations, particularly in younger women [7]. This review aims to discuss the accelerated atherosclerotic risk seen during the menopause transition, highlight the key trial findings on the impact of MHT on cardiovascular disease, and propose a comprehensive, person-centered approach for evaluating cardiovascular risk when initiating MHT. This framework integrates evaluation of both modifiable and non-modifiable CVD risk factors, including universal screening for elevated lipoprotein(a) [Lp(a)] levels, recognition of female-specific CVD risk-enhancers, and leveraging vascular imaging to screen for subclinical vascular disease to support an individualized risk stratification.

## Menopause and MHT Effects on Cardiovascular Risk

While the risk for atherosclerotic cardiovascular disease (i.e. myocardial infarction and stroke) increases with aging, there is an accelerated increase in the incidence of CVD after menopause [8, 9]. The use of MHT for managing menopausal symptoms has a complex relationship with several risk factors for atherosclerosis. Key risk factors and MHT effects include (Table 1):

**Table 1:** Effects of menopause and menopausal hormone therapy on cardiovascular risk factors: A summary of clinical and metabolic changes

Risk Factor	Effect of Menopause	Effect of MHT
Blood Pressure (BP)	- Systolic BP ↑ 4–7 mm Hg - Diastolic ↑ 3–5 mm Hg - Accelerated age-related BP ↑	- Oral estrogen ↓ SBP by 1–6 mm Hg - Combined therapy ↑ SBP - Transdermal estrogen ↓ DBP by up to 5 mm Hg
Weight, Adiposity, and BMI	- ↑ Visceral and pericardial fat - ↑ BMI and waist circumference linked to CHD and mortality	- Modest ↓ visceral fat - ↓ BMI (~ 1 kg/m²) - Preserves lean tissue mass
Insulin Resistance	- ↑ Insulin resistance (OR 1.40–1.59) - ↑ HbA1c by ~ 5%	- ↑ Insulin sensitivity - ↓ HbA1c by up to 0.6% - ↓ Fasting glucose by ~ 20 mg/dL
Lipid Profile Changes (HLD)	- ↑ total cholesterol (10–14%) - ↑ LDL (10–20 mg/dL) - ↑ApoB (8–15%) - ↑ Initially, then ↓ HDL	- ↓ LDL (9–18 mg/dL) - ↑ HDL - Transdermal estrogen is more favorable for triglycerides (less elevation than oral) - No overall CVD risk ↓
Lipoprotein(a) [Lp(a)]	- ↑ by ~ 25% during menopause - ↑ ASCVD risk with Lp(a) > 50 mg/dL - ↑↑ ASCVD risk with Lp(a) > 100 mg/dL (doubled)	- ↓ Lp(a) by 20–30%, oral > other forms - Does not ↓ CVD events
Coronary Artery Calcification (CAC)	- ↑ CAC scores (OR 2.37) Mean CAC = 53	- Oral estrogen ↓ CAC - Transdermal may↑ CAC
Carotid Atherosclerosis	- ↑ CIMT progression - Independent predictor of stroke and CHD	- Early initiation may slow CIMT - No benefit with lower dose or delayed start
Physical Activity, Smoking, and Diet	- ↓ Physical activity - ↓ Dietary quality - No change in smoking status	- No direct effect on physical activity, diet, or smoking habits
Myocardial Infarction (MI) Risk	- ↑ MI risk (accumulation of other CV risk factors)	- CEE + MPA formulation ↑ MI risk (HR 1.29) - Transdermal formulation is safer
Stroke Risk	- ↑ Ischemic stroke risk (HR 1.1–2.0) - ↑↑ in early-onset menopause	- Oral estrogen ↑ stroke risk (~ 40%) - Transdermal < 50 mcg is safer - Risk unaffected by initiation timing

Table 1. Summary of Cardiovascular Risk Factors Affected by Menopause and Menopausal Hormone Therapy (MHT). This table outlines key clinical, laboratory, and imaging-based risk factors associated with menopause, along with the corresponding effects of MHT. Menopause contributes to worsening of blood pressure, central adiposity, insulin resistance, lipid profiles, and subclinical atherosclerosis as measured by CAC and CIMT. MHT demonstrates variable effects depending on formulation and timing, with oral estrogen often improving lipid profiles and Lp(a) levels, but without a clear reduction in cardiovascular events. Transdermal formulations tend to have more favorable effects on blood pressure and metabolic risk, though stroke and thrombotic risks remain formulation- and dose-dependent.

### Vital Signs

- **Blood Pressure (BP)**: Blood pressure tends to increase during menopause. Systolic BP (SBP) increases by 4–7 mm Hg and diastolic BP (DBP) increases by 3–5 mm Hg on average during menopause [10–15]. High blood pressure is the leading preventable risk factor for stroke and myocardial infarction [10–15]. Age-related rises in blood pressure accelerate during menopause [16]. Oral estrogen therapy can lead to minor reductions in SBP by about 1–6 mm Hg [17–20]. However, when combined with progesterone, it leads to small increases in SBP. Transdermal therapy can have neutral or beneficial effects by decreasing DBP up to 5 mm Hg [21, 22].
- **Weight**,** Adiposity**,** and Body Mass Index (BMI)**: While increased weight and BMI are not independently linked to menopause, accelerated visceral adipose tissue and pericardial fat deposition are associated with the loss of ovarian function during menopause [12, 23–25]. Many women in the age range for menopause have an age-adjusted elevated prevalence of obesity compared to younger women. Post-menopausal women with elevated BMI (*≥* 40), waist circumference of greater than 115.5 cm (compared with < 108.4 cm), and central adiposity have increased CHD, heart failure, and mortality [26–28]. Several studies have shown that MHT causes minimal weight gain and is associated with a modest reduction in total and visceral adiposity, smaller waist circumference increases, and lower BMI (approximately 1 kg/m²) compared to no therapy [29–32]. Additionally, MHT preserves lean soft tissue mass that can attenuate the shift toward central adiposity seen after menopause [2].

### Laboratory Values

- **Insulin Resistance**: Menopause often coincides with worsening cardiometabolic health, including insulin resistance and new-onset diabetes, contributing to heightened cardiovascular risk. One study found that post-menopausal women had higher fasting hemoglobin A1c levels by 5% compared to premenopausal women [33]. In one Japanese study, the effects of natural and surgical menopause on insulin resistance were investigated, and the odds ratios (OR) were 1.40 and 1.59, respectively, compared with premenopausal women [34]. MHT can reduce insulin resistance in postmenopausal women when initiated within the early onset of menopause but it depends on the dose and formulation [2, 35, 36]. Meta-analyses have shown that MHT reduces HbA1c (up to 0.6%) and fasting glucose (about 20 mg/dL), as well as improves glucose regulation in women with type 2 diabetes [37].
- **Lipid Profile Changes and Hyperlipidemia (HLD)**: Menopause independently leads to adverse changes in lipid profiles, including elevating total Cholesterol by 10–14%, low-density lipoprotein (LDL) Cholesterol by 10–20 mg/dL or 14–19%, and apolipoprotein B (Apo(B)) levels by 8–15% rather than as a result of aging alone [12, 38–43]. High-density lipoprotein cholesterol (HDL) peaks during peri- and early menopause before declining later in life [38–47]. High HDL in menopause appears to correlate with increased carotid atherosclerosis, the inverse impact seen in the pre-menopause state [5, 48]. It is postulated that the HDL particle’s intrinsic antioxidant and antiatherogenic function weakens during menopause [48, 49]. Overall, menopause is associated with the development of a more atherogenic lipid profile. Oral MHT reduces LDL (by 9–18 mg/dL) and raises HDL, but can raise triglycerides [32, 50, 51]. Transdermal estrogen formulation MHT have less of an effect on triglycerides and LDL [50]. Despite the benefits of MHT on LDL, MHT does not translate to reduced overall CVD risk.
  - **Lipoprotein(a)**: Lp(a) is six times more atherogenic than LDL on a per-particle basis. Elevated Lp(a) (> 50 mg/dL) is associated with an increased 10-year atherosclerotic cardiovascular disease (ASCVD) risk, and the cardiovascular risk of Lp(a) is directly correlated with the degree of Lp(a) elevation [52, 53]. Lp(a) levels are 5–10% higher in women than in men [54]. Across the lifespan, Lp(a) levels are relatively stable in men, while women have an increase in Lp(a) during menopause by approximately 25% [55, 56]. Approximately 90% of Lp(a) levels are determined by genetic factors, but they can also be influenced by diet and hormones [55]. Estrogen use for menopause (both oral and transdermal formulations) has been shown to lower Lp(a) by 20–30%, though this decrease in Lp(a) does not translate to decreased CVD risk [31, 57–59]. This paradox is poorly understood, and further research on the cardio-prognostic effects of Lp(a) lowering is warranted.

### Imaging

- **Coronary Artery Calcification (CAC)**: Increased CAC scores indicate subclinical atherosclerosis and predict future CV events [60, 61]. CAC scores are computed by noninvasive imaging with coronary CT to quantify calcified atherosclerosis burden in the coronary arteries. It can provide robust short- and long-term risk stratification. It can be useful for to further refine ASCVD risk estimation when deciding whether to initiate primary prevention statin therapy. A higher CAC score indicates higher CVD risk in both men and women. A calcium score of zero has a strong negative predictive value for short-term ASCVD events [62]. For context, CAC scores of 1–99 Agatston units carry a 10-year ASCVD risk that varies by age (3.8% for ages 45–54, 6.5% for ages 55–64, and 8.3% for ages 65–74), suggesting moderately increased atherosclerosis risk that can be addressed in an individualized manner [63]. However, CAC scores greater than or equal to 100 Agatston units have a higher 10-year ASCVD risk of more than 7.5%, the widely accepted threshold for initiating statin therapy, regardless of demographic subset, due to a more significant atherosclerosis burden [63]. Additionally, the American College of Radiology (ACR) Guidelines indicate that the estimated 5-year number needed to treat (NNT) for statin therapy to prevent an coronary event is 549 in patients with zero CAC versus 42 in patients with nonzero CAC [64]. Furthermore, those with a CAC score *≥* 100 and elevated Lp(a) appear to be at higher ASCVD risk than those with a similar CAC score (*≥* 100) but normal Lp(a). This data supports that CAC scoring can further define ASCVD risk in those with elevated Lp(a) and help guide primary prevention strategies [65]. Menopause is associated with an elevated CAC *≥* 0, with an OR of 2.37 [66]. According to the Healthy Women Study, a mean CAC score of 53 was reported in post-menopausal women [67, 68]. The degree of CAC has been shown to increase significantly around the menopause transition and indicate subclinical atherosclerosis and CVD risk. For example, women with Type 1 diabetes have been shown to have an approximately 75% increase in CAC volume after menopause, compared to premenopausal values [69]. Therefore, CAC testing should be considered when assessing CV risk in women during menopause. There has been conflicting data on the overall effect of MHT on CAC. In the Women’s Health Initiative (WHI) study, it was found that oral estrogen lowered CAC compared with placebo [70–72]. However, in the Kronos Early Estrogen Prevention Study (KEEPS) study, different types of MHT formulations were tested, and found that synthetic or conjugated equine estrogen (CEE) improved epicardial adipose tissue progression, which is linked to CAC deposition, but that transdermal MHT was linked to increasing CAC progression [73].
- **Carotid Atherosclerosis**: Carotid intima-media thickness (CIMT) is a marker of subclinical atherosclerosis. CIMT is measured using ultrasonography, and elevated CIMT is an independent risk factor for stroke and coronary artery disease [74]. For example, the Atherosclerosis Risk in Communities (ARIC) trial found that increased CIMT (≥ 1.0 mm) had a higher risk of MI and CHD (HR 2.62 in women and 1.20 in men) [75]. CIMT increases at an accelerated rate during late perimenopause [60]. Therefore, CIMT testing can be used to assess patients’ CVD and stroke risk during menopause. While some data suggest benefit of MHT on CIMT progression when initiated early within the perimenopausal period (within 6 years of onset), this effect is not consistently seen in clinical trials [2, 76].

### Lifestyle

- **Physical Activity**,** Cigarette Smoking**,** and Healthy Diet**: These factors are all important components of cardiovascular health for women in the menopausal transition. Data from the Study of Women’s Health Across the Nation (SWAN), a prospective, observational study of women during midlife, found that a healthy lifestyle (including abstinence from smoking, healthy diet, and engagement in regular physical activity) during the menopause transition was associated with less subclinical atherosclerosis later in life [77]. These data emphasize the importance of encouraging a healthy diet, regular physical activity, and avoiding smoking during the menopause transition [78–80].

### Overall CV Risk

- **Myocardial Infarction (MI) Risk**: Menopause increases the risk of MI due to the accumulation of CV risk factors already discussed. MHT, particularly with synthetic formulations of estrogen and progesterone, increases the risk for MI in the general population but may decrease risk when initiated closer to the onset of menopause (before the age of 60 and within 10 years of menopause onset) (HR 1.29, 95% CI 1.02–1.63) [2, 6, 72]. Natural MHT (i.e. estradiol and micronized progesterone) and transdermal estrogen formulations have been shown to have a lower risk of MI than synthetic formulations.
- **Stroke Risk**: Menopause increases the risk of stroke, especially when menopause occurs early (before the age of 45), with a hazard ratio of 1.1 to 2.0 [2, 81, 82]. Both oral estrogen-only and combined MHT increase ischemic stroke risk as demonstrated by the WHI trial. Transdermal estrogen, especially at doses < 50 mcg, appears to have lower stroke risk based on observational data. The risk of stroke remains elevated no matter the timing of initiation of oral MHT [6, 81, 83].

## Types of MHT, Delivery Methods, and CVD Risk

MHT is a tool that providers may use to address some of the symptoms of menopause that may negatively impact patients’ quality of life. The formulation and delivery of MHT significantly influence its risk profile for CVD, including venous thromboembolism, stroke, and CAD. There is significant literature, including large, randomized controlled data, that assesses the vascular risks of oral MHT formulation. Most literature on transdermal MHT formulation is based on an observational study.

- **Systemic Estrogen and Progesterone**: Combined MHT (estrogen and progesterone) carries a higher CVD risk than estrogen-only MHT [70], suggesting that progesterone plays a role in adverse outcomes, including elevated stroke and venous thromboembolism risk with elevation of very low density lipoprotein (VLDL) and C-reactive protein (CRP) [84]. Medroxyprogesterone acetate (MPA) has been shown to attenuate the beneficial LDL-lowering effects of estrogen [85]. A seminal study discussed later in this review, the WHI, also found that CEE and MPA, both synthetic forms of estrogen and progesterone, respectively, demonstrate elevated CVD risk in older postmenopausal women, though prescription of synthetic hormones has fallen significantly out of favor due to their overall increased CVD risk [6, 70, 71, 86]. Women who possess a uterus require progesterone to mitigate the risk of endometrial hyperplasia with unopposed estrogen. Oral estrogen has been found to decrease LDL by 15–19%, increase HDL by 16–18%, and increase triglycerides by 24–42% [87]. Additionally, oral estrogen has favorable effects on insulin resistance and blood pressure [85]. Estrogen has been shown to lower CAC progression (OR 0.78, 0.74, and 0.69 for CAC scores of 0, 10 or more, and 100 or more, respectively) [71]. However, despite these benefits on individual risk factors, estrogen has not been shown to have an overall reduction in cardiovascular events and has been shown to have significant thrombotic risks [85].
  - **Timing of Initiation**: Women who are initiated on systemic MHT closer to the onset of menopause have been found to have less CHD risk than those more than 10 years from menopause [85].
- **Dose Dependent Relationship**: Lower dose estrogen dosing (0.5 mg oral estradiol) compared with conventional or high dose estrogen (1–2 mg oral estradiol) has lower cardiovascular risk [88].
- **Natural vs. Synthetic Estrogen**: There appear to be higher risks of venous thromboembolism (VTE) and possible MI with synthetic estrogen (CEE, ethinyl estradiol) compared with natural estrogen (estradiol) use [88–90].
- **Natural vs. Synthetic Progesterone**: Older synthetic progesterone formulations (MPA, Norethindrone acetate, levonorgestrel, drospirenone), as used in the WHI and Heart and Estrogen/Progestin Replacement Study (HERS) studies, can lower HDL by 8–18%. Natural progesterone (micronized progesterone), in contrast, has a neutral impact on HDL. Natural progestin also had a lower VTE risk compared with synthetic progestin [85, 89, 91].
- **Bioidenticals (Natural)**: Bioidentical hormones are derived from plant extracts and are modified to be structurally identical to endogenous estrogen (such as estradiol and micronized progesterone). In society, when referencing bioidentical hormones, people may be referring to custom-compounded, multi-hormone regimens that are dose-adjusted based on serial hormone levels. Compounded bioidentical hormone therapy (cBHT) has variability in quality, consistency, and relatable dosing. CBHT also lacks rigorous quality control, which can affect potency, purity, and absorption, leading to inconsistent therapeutic effects and possible safety concerns. Similarly, no significant clinical data support the efficacy or long-term safety of cBHT [92]. Therefore, the Menopause Society, American College of Obstetricians and Gynecologists, and Endocrine Society advise against cBHT. Furthermore, pellet formulations are not advised due to unpredictable bioavailability [92].
- **Transdermal Estrogen**: Transdermal formulations are associated with lower thrombotic and stroke risks compared to oral estrogen, making them a preferred option for patients with dyslipidemia or hypertriglyceridemia, but have no direct effect on coronary atherosclerosis [89]. However, these findings are based on smaller observational studies because, to date, no direct randomized control trial (RCT) compares oral and transdermal therapy to stroke/VTE risk. There is a dose-dependent relationship in transdermal estrogen prescription as well, and lower dose formulations should be pursued to minimize CVD risk.
- **Vaginal Estrogen Therapy**: While oral and transdermal MHT address vasomotor symptoms given systemic effects, vaginal estrogen therapy addresses genitourinary symptoms including vulvovaginal atrophy, painful intercourse, and frequent urinary tract infections. Low-dose vaginal estrogen therapy is most effective for these symptoms which typically increase as patient’s increase with age after menopause. Vaginal estrogen therapy has minimal systemic absorption and therefore is not associated with heightened cardiovascular risk, and as a result may be useful for patients with contraindications for systemic MHT [90–93].

## Clinical Trials and Evidence on MHT and CVD

The trials below represent the key literature in understanding the history of MHT on cardiovascular risk.

- **Women’s Health Initiative (WHI)**: WHI studied the primary prevention of MHT and found increased CVD events in older women randomized to MHT [70]. This study was a large-scale, multi-center, RCT that enrolled 161,808 postmenopausal women aged 50–79 from 1993 to 1998 across 40 centers in the USA and tested MHT on 27,347 patients. This was an older, postmenopausal population, with a mean of 63 years, with the majority enrolled greater than 10 years since menopause, and did not have vasomotor symptoms [94]. The two main hormonal interventions included (1) oral CEE (0.625 mg/day) + MPA (2.5 mg/day) vs. placebo, and (2) oral CEE (0.625 mg/day) alone in women who had undergone prior hysterectomy vs. placebo. Primary outcomes were coronary heart disease and invasive breast cancer, with secondary outcomes including stroke, pulmonary embolism (PE), colorectal cancer, endometrial cancer, hip fracture, and death from other causes. Notably, in those treated with CEE + MPA, there was an increased risk of CHD (HR 1.29, 95% CI 1.02–1.63) and stroke (HR 1.41, 95% CI 1.07–1.85). Further subgroup analysis also demonstrated who with higher LDL had higher CHD risk while on CEE + MPA. Subgroup analysis of the CEE + MPA groups showed that the increased CHD risk was seen primarily in those who were older and further from the menopause transition (*≥* 10 years from menopause). Stroke risk similarly increased with age in those treated in CEE + MPA, however, the stroke risk remained elevated across age groups, including those near menopause [72, 95]. In contrast to those treated with CEE + MPA, those treated with CEE alone did not have increased CHD risk (HR 0.94, 95% CI 0.78–1.14), but increased stroke risk was seen (1.39, 95% CI 1.10–1.77) [6]. Given the difference in CHD outcomes between the CEE + MPA and CEE-alone groups, the MPA was believed to contribute to the increased CHD risk. Subgroup analyses of both groups found that CAC and Lp(a) were reduced with estrogen therapy, but that stroke risk was increased [6, 71, 72, 86, 96, 97]. Hazard ratio findings are detailed in Table 2.

**Table 2:** Relative risk of major health outcomes in the women’s health initiative: comparison of conjugated equine Estrogen plus Medroxyprogesterone acetate (CEE + MPA) vs. Estrogen alone (CEE)

Outcome	CEE + MPA (HR, 95% CI)	CEE Alone (HR, 95% CI)
Coronary Heart Disease	1.29 (1.02–1.63)	0.94 (0.78–1.14)
Invasive Breast Cancer	1.26 (1.00-1.59)	0.79 (0.61–1.02)
Stroke	1.41 (1.07–1.85)	1.39 (1.10–1.77)
PE	2.13 (1.39–3.25)	1.34 (0.87–2.06)
Colorectal Cancer	0.63 (0.43–0.92)	1.08 (0.75–1.55)
Endometrial Cancer	0.83 (0.47–1.47)	Not applicable (hysterectomy)
Hip Fracture	0.66 (0.45–0.98)	0.61 (0.41–0.91)
Death from Other Causes	0.98 (0.82–1.18)	1.04 (0.88–1.22)

Table 2. Clinical Outcomes Associated with Menopausal Hormone Therapy in the Women’s Health Initiative (WHI). This table summarizes hazard ratios (HR) with 95% confidence intervals for key clinical outcomes in postmenopausal women randomized to conjugated equine estrogen plus medroxyprogesterone acetate (CEE + MPA) versus estrogen alone (CEE) in the WHI trial. CEE + MPA was associated with increased risk of coronary heart disease, invasive breast cancer, stroke, and pulmonary embolism, while reducing the risk of hip and colorectal cancers. Estrogen-only therapy showed a neutral effect on coronary heart disease and was not associated with increased breast cancer risk, but it similarly increased stroke risk. Endometrial cancer data were not applicable in the estrogen-only arm due to prior hysterectomy. These results underscore the importance of individualized risk assessment when considering hormone therapy.

- **Heart and Estrogen/Progestin Replacement Study (HERS)**: HERS I and HERS II were secondary prevention trials for women who already had known heart disease (CAD confirmed by angiography, MI history, coronary artery bypass graft (CABG)) [98, 99]. The study enrolled 2763 postmenopausal women, on average 67 years old, with CVD from 20 centers across the nation. Participants were randomly assigned to receive synthetic CEE (0.625 mg/day) + MPA (2.5 mg/day) vs. placebo. HERS I was randomized, double blind, and placebo-controlled, followed over 4.1 years, while HERS II was an open-label extension with an additional 2.7 years after HERS I [98]. The primary outcome was the occurrence of nonfatal myocardial infarction (MI) or CHD death. HERS I reported increased CHD events in the first year of MHT (HR 1.52, 95% CI 1.01–2.29), while HERS II found no sustained benefit in CHD risk reduction over time up to 6.8 years (HR 0.99, 95% CI 0.84–1.17), with continued increased VTE risk (HR 2.89, 95% CI 1.50–5.58) [100]. A subgroup analysis of this trial found that the increased CHD risk during the first year of MHT use in those with established CHD was not seen in those on statin therapy [77].
- **The Early versus Late Intervention Trial with Estradiol (ELITE) Trial and Kronos Early Estrogen Prevention Study (KEEPS)**: The ELITE and KEEPS Trials examined the “timing/endothelium hypothesis,” which posits that the effect of MHT on the endothelium differ depending on the timing of initiation relative to the onset of menopause. MHT may have cardioprotective effects on a healthy endothelium; however, MHT may have detrimental effects on a diseased endothelium with established atherosclerotic plaques [101]. The ELITE trial demonstrated that oral estradiol reduced the progression of CIMT if initiated less than 6 years after menopause (*p* = 0.008) [102]. The population was 643 healthy postmenopausal women without diabetes or CVD who were stratified into early (< 6 years) or late (> 10 years) post-menopause. They were randomized to receive either oral 17β-estradiol (1 mg/day) vs. placebo if the patients had no uterus and vaginal micronized progesterone (45 mg/day for 10 days each month) was added vs. placebo for patients with a uterus. In contrast, the KEEPS trial similarly studied the timing of initiation of MHT but studied synthetic oral estrogen and transdermal estrogen [76]. This population was 727 healthy women within 3 years of initiating menopause who were randomized to receive oral synthetic estrogen (CEE 0.45 mg/day) + micronized progesterone (200 mg/day for 12 days each month), or transdermal 17β-estradiol (t-E2, 50 µg/day) + micronized progesterone (200 mg/day for 12 days each month), vs. placebo. The primary outcome studied was the progression of CIMT, but no significant effect of either intervention was shown. An additional outcome studied was a trend for reduced CAC with CEE, but this was not statistically significant. Overall, research findings on the potential CV protective benefits of early MHT initiation are conflicting, and further research evaluating the timing, dose, and formulation effects on CV health during the menopause transition is needed [2].

In summary, MHT is not recommended for the primary or secondary prevention of cardiovascular disease [103]. The HERS trials of MHT in postmenopausal women with established CVD did not find MHT to be cardio-protective, and in HERS showed a signal of harm in the first year of initiation. The findings from WHI demonstrated that MHT (in the form of CEE + MPA) does not confer protection for the primary prevention of CHD and may increase the risk of CHD when initiated in older postmenopausal women, especially during the first year after the initiation of hormone use. Furthermore, in WHI, CEE, and MPA, increased stroke risk, regardless of age or years since menopause. CEE alone does not appear to increase CHD risk in the population, but does increase stroke risk regardless of years from menopause onset, and these risks heighten with age. Notably, WHI was a study of older, post-menopausal women who were not experiencing significant menopause symptoms. Today, most patients who are being considered for MHT to alleviate symptoms are near the menopause transition and often 10–15 years younger than most of the population studied in WHI. Evidence suggests that women in early menopause who are in good cardiovascular health are at low risk of adverse cardiovascular outcomes and should be considered candidates for the use of MHT for relief of menopausal symptoms [103]. Therefore, when initiating MHT, it is important to consider the patient’s age, years from the onset of menopause, and risks for cardiovascular events to appropriately counsel the patient on the potential CV risks of MHT.

## Comprehensive Approach To CV Risk Assessment when Initiating MHT Assessment

**Fig. 1:**
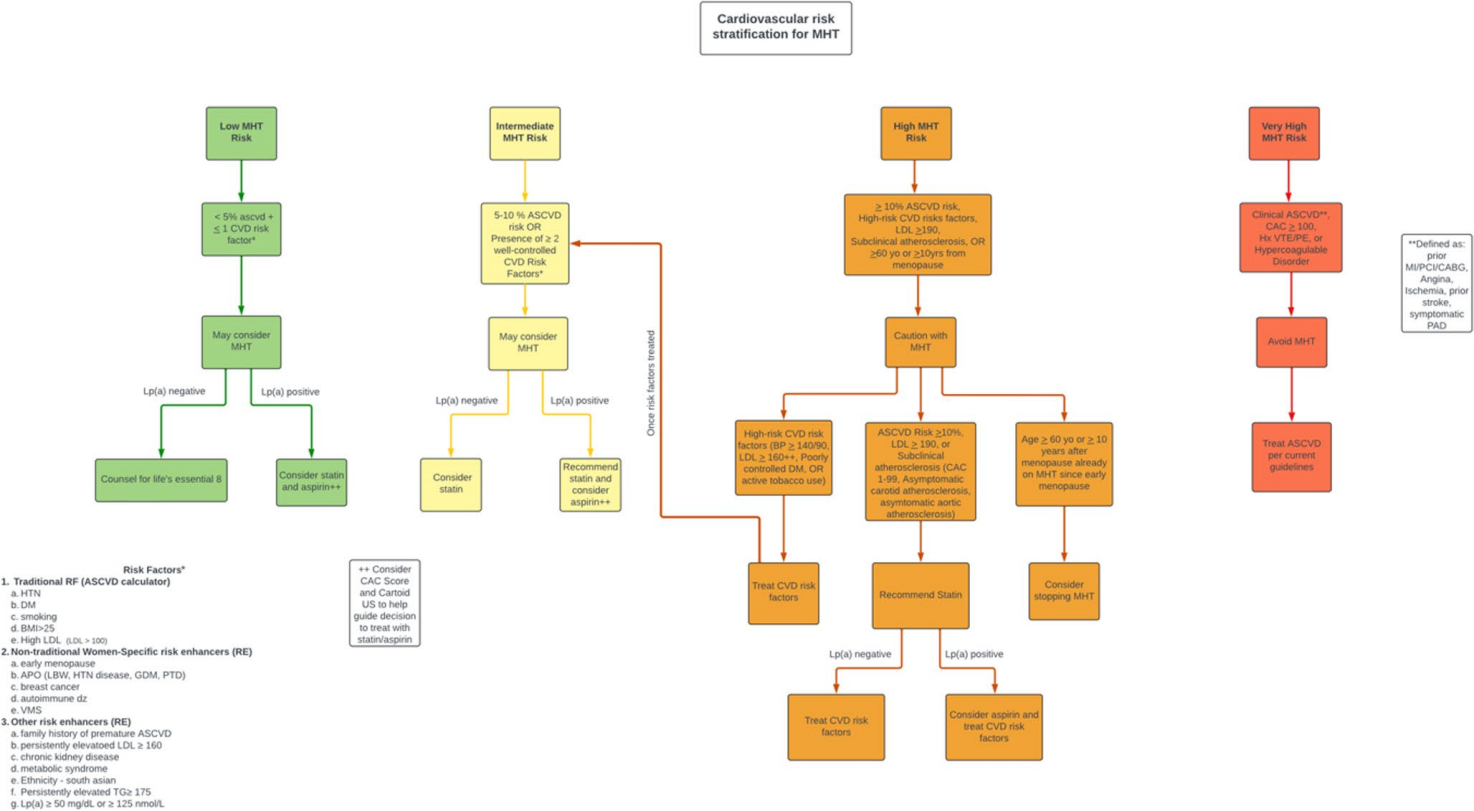
Risk-Based Algorithm for Guiding Menopausal Hormone Therapy Using ASCVD Score, Subclinical Atherosclerosis, and Lp(a) Stratification. Cardiovascular Risk Stratification for Menopausal Hormone Therapy (MHT). This clinical decision tool integrates traditional ASCVD risk scoring, presence of cardiovascular disease (CVD) risk factors, and subclinical atherosclerosis to guide MHT use. Patients are stratified into low, intermediate, high, or very high risk based on ASCVD estimates, comorbidities, imaging findings (CAC, carotid ultrasound), and history of thrombotic disease. MHT is generally considered appropriate for low- and select intermediate-risk individuals. Caution is advised in high-risk patients, and MHT should be avoided in very high-risk patients, including those with clinical ASCVD and CAC *≥* 100 AU. Statin and aspirin therapy may be considered based on ASCVD risk score, Lp(a) levels, and imaging results. This framework supports individualized, risk-informed decision-making in midlife women. Created in MSVisio. Spertus, E. (2025)

Multiple recent algorithms are available when assessing CV risk and initiating MHT. The fundamental steps include: (1) perform a person-centered CV risk assessment, including the presence of known MHT contraindications, age and time since menopause, and severity of menopause symptoms, (2) provide a recommendation for MHT use and formulation based on CV risk category, (3) ensure to reassess risks versus benefits of MHT at follow-up [104, 105]. This proposed algorithm incorporates universal Lp(a) screening and consider the presence of subclinical atherosclerosis in risk assessment. Additionally, this framework highlights the consideration of MHT formulation and CV risk prevention strategies while on MHT. In the proposed algorithm (Fig. 1), menopausal women are stratified into four risk categories—low, intermediate, high, and very high—based on their ASCVD risk score, presence of CV risk factors, and evidence of subclinical or clinical atherosclerosis .

1. Perform Comprehensive Person-Centered CV Risk Assessment
  **ASCVD Risk Score**: The ASCVD risk score is a tool to estimate an individual’s 10-year risk of developing cardiovascular events like MI and stroke, incorporating several factors such as age, sex, race, total cholesterol, HDL cholesterol, systolic blood pressure, diabetes, and smoking status to predict risk [106]. The ASCVD risk score is used to determine who may benefit from statin therapy for the primary prevention of ASCVD [107]. Furthermore, a patient’s ASCVD risk assessment can be considered to assess their atherosclerotic risk and potential risk for atherosclerotic complications from MHT [108].
  **ASCVD Risk Enhancing Factors**: Many risk factors for CV disease are not included in the ASCVD risk score calculation. Examples include family history of premature ASCVD, chronic auto-inflammatory conditions (i.e. lupus, rheumatoid arthritis, human immunodeficiency virus), and several biomarkers, including elevated high-sensitivity C-reactive protein, elevated Lp(a), and elevated Apolipoprotein (B) [107]. Furthermore, there are several female-specific risk factors for ASCVD, including a history of premature menopause, polycystic ovarian syndrome, and hypertensive disorders of pregnancy. As discussed above, evaluation of subclinical atherosclerosis (CAC scoring, carotid ultrasound) can be used to further refine a patient’s atherosclerotic risk estimation and should be considered when assessing risk prior to starting MHT. Therefore, when estimating ASCVD risk for the initiation of MHT, it is important to consider that these risk-enhancing factors should be incorporated into the risk assessment [109].
  **Thromboembolic Risk Enhancing Factors**: The presence of hypercoagulable disorders (i.e. Factor V Leiden) or a personal history of VTE or PE should be considered when assessing thrombotic risks associated with MHT use.
2. Provide a Recommendation for MHT Use and Formulation Based on CV Risk Category
  **Low Risk**: Women with low risk for CV events related to MHT include women who are less than 60 and within 10 years of menopause, who have a low estimated ASCVD risk (< 5%), and one or fewer CV risk factors, and can be considered for MHT prescription. All patients, regardless of risk, should be counseled on a heart-healthy lifestyle given the accelerated CV risk seen with menopause. Those with elevated Lp(a) (≥ 50 mg/dL or ≥ 125 nmol/L) should be considered for statin and aspirin therapy while on MHT to lower CV risk. While there are currently no approved medications approved for the indication to lower Lp(a), the current recommended management for elevated Lp(a) is optimization of LDL levels (including lifestyle and statin therapy), and other modifiable CV risk factors (i.e., hypertension, diabetes). The degree of Lp(a) should be considered when initiation primary prevention treatment. An Lp(a) level *≥* 180 mg/dL is an extreme elevation and carries an increased lifetime risk of ASCVD similar to those with heterozygous familial hyperlipidemia. Aspirin, which reduces platelet activation, may help offset the prothrombotic properties of Lp(a) [110, 111]. Several recent studies have suggested that aspirin may be beneficial for the primary prevention of CV events in those with elevated Lp(a) [111, 112]. Per the 2019 ACC/AHA Primary Prevention Guidelines, aspirin 81 mg may be considered for the primary prevention of ASCVD in select adults aged 40 to 70 who are at increased risk of ASCVD but not increased risk of bleeding [107]. CAC scoring and carotid ultrasound can help guide whether to initiate a statin or aspirin in patients with elevated Lp(a) while on MHT.
  **Intermediate Risk**: Intermediate risk includes women with 5–10% ASCVD risk or the presence of one to two well-controlled CV risk factors. Similarly, these patients are considered lower risk with MHT, but statin and aspirin use may be considered in the population, particularly if risk-enhancing factors are present, like elevated Lp(a). Furthermore, an intermediate risk patient should consider transdermal estrogen formulation and micronized progesterone, given the more favorable CV risk compared to oral estrogen and synthetic progesterone.
  **High Risk**: High risk encompasses those with estimated high (> 10%) ASCVD risk, uncontrolled high-risk CVD risk factors, evidence of subclinical atherosclerosis (such as CAC 1–99 or asymptomatic carotid plaques), and older postmenopausal women (*≥* 60 years old or *≥* 10 years from menopause). This higher-risk population should be counseled to be cautious with MHT and consider non-MHT therapy. Their cardiovascular risk factors should be treated, and they may potentially transition to a lower risk category with treatment. However, MHT is not contraindicated in this group and may be considered to alleviate menopause symptoms after thorough shared decision making with the patient and their provider. Favored MHT formulation for this group includes a low-dose transdermal estrogen formulation and micronized progesterone.
  **Very High Risk**: Very high risk is defined by the presence of clinical ASCVD (e.g., prior MI, stroke, or PAD), CAC > 100, or high-clot risk (like prior VTE or certain hypercoagulable disorders). In this group, we advise patients to avoid systemic MHT, but non-MHT therapies for menopause symptoms should be explored. These patients should still be considered for vaginal estrogen therapy given limited systemic effects. Patients with a history of spontaneous coronary artery dissection (SCAD) should avoid MHT, given that estrogen plays a key role in the risk of dissection [113, 114]. Those with fibromuscular dysplasia (FMD) should be counseled on the potential vascular risk of MHT and should explore non-MHT treatment for menopause symptoms [115].

3) Reassess Risks of MHT at Follow-up.

After initiating MHT, it is important to repeat the CV risk assessment at regular follow-up. As patients age, and their cardiovascular risks increase, it is important to consider when to discontinue MHT based on ongoing risk and benefit conversations.

## Conclusions and Future Directions

In summary, the menopause transition is a critical window for care and individual cardiovascular assessment due to the rise of risk factors for CVD inherent during this time. Though many interventions of MHT can alleviate the uncomfortable symptoms of menopause they may also augment cardiovascular risk. Therefore, personalized assessment of ASCVD risk and benefit analysis prior to initiation of MHT is necessary during this time. While there are many benefits of utilizing this approach, it is important to note some of the limitations of the aggregate data. For one, much of the data referenced is from older clinical trials (e.g., WHI, HERS) and references older postmenopausal women (> 63 years) who were not initiating MHT for vasomotor symptoms. This review applies these findings to younger women closer to menopause for limited time courses. Additionally, while contemporary data shows that transdermal estrogen and micronized progesterone MHT formulations have lower cardiovascular risks than oral and synthetic formulations, the supporting evidence is predominantly observational without significant randomized control data. Therefore, MHT formulation should be chosen carefully prior to initiation. Similarly, there is limited data on the risks of initiating MHT in smaller high-risk populations such as those with SCAD or FMD, and this risk is based on pathophysiological rationale and small cohort data. While several national and international cardiology societal guidelines support universal screening for elevated Lp(a), this is not yet implemented routinely in preventative screening assessments. While observational data and secondary analysis from primary prevention aspirin randomized trials support the potential benefit of aspirin in those with elevated Lp(a), randomized clinical trials evaluating the use of aspirin for the primary prevention of cardiovascular disease are needed to better understand the benefit of aspirin while balancing bleeding risks [116]. 

This review presents a comprehensive, person-centered risk approach for evaluating cardiovascular risk in the context of MHT. This framework integrates evaluation of both modifiable and non-modifiable CVD risk factors, including universal screening for elevated lipoprotein(a) (Lp(a)) levels, recognition of female-specific CVD risk-enhancers, and leveraging vascular imaging to screen for subclinical vascular disease to support an individualized risk stratification. The tool proposed builds on existing risk assessment design (such as the ASCVD risk assessment) to estimate ASCVD risk and guide prevention strategies specifically for initiating MHT, while historical tools were not developed or validated for this precise purpose. This tool incorporates specific cardiac markers such as routine Lp(a) measurement at menopause, and assessment for subclinical atherosclerosis (i.e. CAC scoring, carotid ultrasound) to help guide individualized provider-patient shared decision making. Menopausal management draws upon the specialties of Obstetrics/Gynecology, Endocrinology, Cardiology, and Primary Care, and a thorough multidisciplinary approach is warranted in decision making. There has been significant research on MHT and cardiovascular risk, but questions remain for new therapies. Novel therapies such as Lp(a)-targeted therapies may address these specific risk factors, marking a new frontier in managing menopausal cardiovascular health.

## Key References

- Sobel TH, Shen W. Transdermal estrogen therapy in menopausal women at increased risk for thrombotic events: a scoping review. Menopause. 2022;29(4):483–490. 10.1097/GME.0000000000001938.
  - This scoping review highlights the safety of transdermal estrogen as a preferred formulation for women with thrombotic risk, informing personalized MHT strategies.
- Ferreira Campos L, de Andrade Costa G, Domingues Feitosa M, et al. Effect of hormone therapy on blood pressure and hypertension in postmenopausal women: a systematic review and meta-analysis. Menopause. 2024;31(6):556–562. 10.1097/GME.0000000000002359.
  - This meta-analysis provides updated evidence on how MHT influences blood pressure, critical for cardiovascular risk assessment.
- Jiang X, Aragaki AK, Nudy M, et al. The association of hormone therapy with blood pressure control in postmenopausal women with hypertension: a secondary analysis of the Women’s Health Initiative. Menopause. 2023;30(1):28–36. 10.1097/GME.0000000000002086.
  - Supports the nuanced effect of MHT on hypertension depending on formulation and timing.
- Speksnijder EM, Ten Noever de Brauw GV, Malekzadeh A, et al. Effect of Postmenopausal Hormone Therapy on Glucose Regulation in Women With Type 1 or Type 2 Diabetes: A Systematic Review and Meta-analysis. Diabetes Care. 2023;46(10):1866–1875. 10.2337/dc23-0451.
  - This meta-analysis demonstrates the glycemic benefits of MHT, particularly relevant for women with diabetes.
- Torosyan N, Visrodia P, Torbati T, et al. Dyslipidemia in midlife women: Approach and considerations during the menopausal transition. Maturitas. 2022;166:14–20. 10.1016/j.maturitas.2022.08.001.
  - A key article summarizing lipid changes during menopause and guiding dyslipidemia management in this population.
- Nie G, Yang X, Wang Y, et al. The Effects of Menopause Hormone Therapy on Lipid Profile in Postmenopausal Women: A Systematic Review and Meta-Analysis. Front Pharmacol. 2022;13:850815. 10.3389/fphar.2022.850815.
  - Provides detailed evidence of the favorable effects of MHT on LDL and HDL levels.
- Björnson E, Adiels M, Taskinen MR, et al. Lipoprotein(a) Is Markedly More Atherogenic Than LDL: An Apolipoprotein B-Based Genetic Analysis. J Am Coll Cardiol. 2024;83(3):385–395. 10.1016/j.jacc.2023.10.039.
  - Establishes the high atherogenicity of Lp(a), reinforcing its importance in menopausal CV risk assessment.
- Volgman AS, Koschinsky ML, Mehta A, Rosenson RS. Genetics and Pathophysiological Mechanisms of Lipoprotein(a)-Associated Cardiovascular Risk. J Am Heart Assoc. 2024;13(12):e033654. 10.1161/JAHA.123.033654.
  - Explains how genetic and hormonal factors, including menopause, contribute to Lp(a)-mediated cardiovascular risk.
- Mehta A, Vasquez N, Ayers CR, et al. Independent Association of Lipoprotein(a) and Coronary Artery Calcification With Atherosclerotic Cardiovascular Risk. J Am Coll Cardiol. 2022;79(8):757–768. 10.1016/j.jacc.2021.11.058.
  - Shows Lp(a)'s independent contribution to coronary artery calcification, a pivotal imaging marker in midlife risk assessment.
- Bushnell C, Kernan WN, Sharrief AZ, et al. 2024 Guideline for the Primary Prevention of Stroke: A Guideline From the AHA/ASA. Stroke. 2024;55(12):e344–e424. 10.1161/STR.0000000000000475.
  - Essential guideline outlining stroke prevention approaches, including sex-specific recommendations relevant to MHT.
- Cho L, Kaunitz AM, Faubion SS, et al. Rethinking Menopausal Hormone Therapy: For Whom, What, When, and How Long? Circulation. 2023;147(7):597–610. 10.1161/CIRCULATIONAHA.122.061559.
  - A high-impact summary of how modern evidence should reshape individualized MHT use in clinical practice.
- Nudy M, Aragaki AK, Jiang X, et al. Long-Term Changes to Cardiovascular Biomarkers After Hormone Therapy in the WHI. Obstet Gynecol. 2025;145(4):357–367. 10.1097/AOG.0000000000005862.
  - Evaluates the durable biomarker changes post-MHT, supporting a deeper understanding of long-term cardiovascular risk.
- Hirsch H, Manson JE. Menopausal symptom management in women with cardiovascular disease or vascular risk factors. *Maturitas*. 2022;161:1–6. doi:10.1016/j.maturitas.2022.01.016
  - Provides a summary of best practices when treating menopausal symptoms in women with pre-existing CVD, including addressing risks, formulation, dosing and route of delivery of MHT.

## Data Availability

No datasets were generated or analysed during the current study.

## References

[1] Promoting Risk Identification and Reduction of Cardiovascular Disease in Women Through Collaboration. With obstetricians and gynecologists: A presidential advisory from the American heart association and the American college of obstetricians and gynecologists. 10.1161/CIR.0000000000000582. 10.1161/CIR.0000000000000582 29748185

[2] El Khoudary SR, Aggarwal B, Beckie TM, et al. Menopause transition and cardiovascular disease risk: implications for timing of early prevention: a scientific statement from the American Heart Association. Circulation. 2020;142(25):e506–32. 10.1161/CIR.0000000000000912. 33251828 10.1161/CIR.0000000000000912

[3] Williams RE, Kalilani L, DiBenedetti DB, Zhou X, Fehnel SE, Clark RV. Healthcare seeking and treatment for menopausal symptoms in the United States. Maturitas. 2007;58(4):348–58. 10.1016/j.maturitas.2007.09.006. 17964093 10.1016/j.maturitas.2007.09.006

[4] Menopause Transition and Cardiovascular Disease Risk. Implications for timing of early prevention: A scientific statement from the American heart association. 10.1161/CIR.0000000000000912. 10.1161/CIR.0000000000000912 33251828

[5] El Khoudary SR, Greendale G, Crawford SL, et al. The menopause transition and women’s health at midlife: a progress report from the study of women’s health across the Nation (SWAN). Menopause. 2019;26(10):1213–27. 10.1097/GME.0000000000001424. 31568098 10.1097/GME.0000000000001424 PMC6784846

[6] Pinkerton JV. Hormone therapy for postmenopausal women. N Engl J Med. 2020;382(5):446–55. 10.1056/NEJMcp1714787. 31995690 10.1056/NEJMcp1714787

[7] Sobel TH, Shen W. Transdermal estrogen therapy in menopausal women at increased risk for thrombotic events: a scoping review. Menopause N Y N. 2022;29(4):483–90. 10.1097/GME.0000000000001938. 10.1097/GME.0000000000001938 35357370

[8] Kannel WB, Hjortland MC, McNamara PM, Gordon T. Menopause and risk of cardiovascular disease: the Framingham study. Ann Intern Med. 1976;85(4):447–52. 10.7326/0003-4819-85-4-447. 970770 10.7326/0003-4819-85-4-447

[9] Lundberg G, Wu P, Wenger N. Menopausal hormone therapy: a comprehensive review. Curr Atheroscler Rep. 2020;22(8):33. 10.1007/s11883-020-00854-8. 32556827 10.1007/s11883-020-00854-8

[10] Lejsková M, Alušík S, Valenta Z, Adámková S, Piťha J. Natural postmenopause is associated with an increase in combined cardiovascular risk factors. Physiol Res. 2012;61(6):587–96. 10.33549/physiolres.932313. 23098660 10.33549/physiolres.932313

[11] Janssen I, Powell LH, Crawford S, Lasley B, Sutton-Tyrrell K. Menopause and the metabolic syndrome: the study of women’s health across the Nation. Arch Intern Med. 2008;168(14):1568–75. 10.1001/archinte.168.14.1568. 18663170 10.1001/archinte.168.14.1568 PMC2894539

[12] Thurston RC, Karvonen-Gutierrez CA, Derby CA, El Khoudary SR, Kravitz HM, Manson JE. Menopause versus chronologic aging: their roles in women’s health. Menopause N Y N. 2018;25(8):849–54. 10.1097/GME.0000000000001143. 10.1097/GME.0000000000001143 30045364

[13] Freeman EW, Sammel MD. Methods in a longitudinal cohort study of late reproductive age women: the Penn ovarian aging study (POAS). Womens Midlife Health. 2016;2:1. 10.1186/s40695-016-0014-2. 30766699 10.1186/s40695-016-0014-2 PMC6299955

[14] Gurka MJ, Vishnu A, Santen RJ, DeBoer MD. Progression of metabolic syndrome severity during the menopausal transition. J Am Heart Assoc. 2016;5(8):e003609. 10.1161/JAHA.116.003609. 27487829 10.1161/JAHA.116.003609 PMC5015287

[15] Zanchetti A, Facchetti R, Cesana GC, et al. Menopause-related blood pressure increase and its relationship to age and body mass index: the SIMONA epidemiological study. J Hypertens. 2005;23(12):2269–76. 10.1097/01.hjh.0000194118.35098.43. 16269969 10.1097/01.hjh.0000194118.35098.43

[16] Burt VL, Whelton P, Roccella EJ, et al. Prevalence of hypertension in the US adult population. Results from the third National health and nutrition examination survey, 1988–1991. Hypertension. 1995;25(3):305–13. 10.1161/01.hyp.25.3.305. 7875754 10.1161/01.hyp.25.3.305

[17] Seely EW, Walsh BW, Gerhard MD, Williams GH. Estradiol with or without progesterone and ambulatory blood pressure in postmenopausal women. Hypertension. 1999;33(5):1190–4. 10.1161/01.HYP.33.5.1190. 10334810 10.1161/01.hyp.33.5.1190

[18] Cacciatore B, Paakkari I, Hasselblatt R, et al. Randomized comparison between orally and transdermally administered hormone replacement therapy regimens of long-term effects on 24-hour ambulatory blood pressure in postmenopausal women. Am J Obstet Gynecol. 2001;184(5):904–9. 10.1067/mob.2001.111246. 11303197 10.1067/mob.2001.111246

[19] Ferreira Campos L, de Andrade Costa G, Domingues Feitosa M, et al. Effect of hormone therapy on blood pressure and hypertension in postmenopausal women: a systematic review and meta-analysis. Menopause N Y N. 2024;31(6):556–62. 10.1097/GME.0000000000002359. 10.1097/GME.0000000000002359 38688468

[20] Jiang X, Aragaki AK, Nudy M, et al. The association of hormone therapy with blood pressure control in postmenopausal women with hypertension: a secondary analysis of the women’s health initiative clinical trials. Menopause. 2023;30(1):28–36. 10.1097/GME.0000000000002086. 36256926 10.1097/GME.0000000000002086

[21] Ichikawa J, Sumino H, Ichikawa S, Ozaki M. Different effects of transdermal and oral hormone replacement therapy on the renin-angiotensin system, plasma bradykinin level, and blood pressure of normotensive postmenopausal women. Am J Hypertens. 2006;19(7):744–9. 10.1016/j.amjhyper.2005.10.006. 16814131 10.1016/j.amjhyper.2005.10.006

[22] Kaya C, Cengiz SD, Cengiz B, Akgun G. Long-term effects of low-dose 17beta-estradiol plus dydrogesterone on 24-h ambulatory blood pressure in healthy postmenopausal women: a 1-year, randomized, prospective study. Gynecol Endocrinol Off J Int Soc Gynecol Endocrinol. 2007;23(Suppl 1):62–7. 10.1080/09513590701584956. 10.1080/09513590701584956 17943541

[23] Matthews KA, Abrams B, Crawford S, et al. Body mass index in mid-life women: relative influence of menopause, hormone use, and ethnicity. Int J Obes. 2001;25(6):863–73. 10.1038/sj.ijo.0801618. 10.1038/sj.ijo.0801618 11439301

[24] Davis SR, Castelo-Branco C, Chedraui P, et al. Understanding weight gain at menopause. Climacteric. 2012;15(5):419–29. 10.3109/13697137.2012.707385. 22978257 10.3109/13697137.2012.707385

[25] Rosito GA, Massaro JM, Hoffmann U, et al. Pericardial fat, visceral abdominal fat, cardiovascular disease risk factors, and vascular calcification in a community-based sample: the Framingham Heart Study. Circulation. 2008;117(5):605–13. 10.1161/CIRCULATIONAHA.107.743062. 18212276 10.1161/CIRCULATIONAHA.107.743062

[26] Ogden CL, Carroll MD, Fryar CD, Flegal KM. Prevalence of obesity among adults and youth: united states, 2011–2014. NCHS Data Brief. 2015;(219):1–8. 26633046

[27] McTigue KM, Chang YF, Eaton C, et al. Severe obesity, heart disease, and death among white, African American, and Hispanic postmenopausal women. Obesity. 2014;22(3):801–10. 10.1002/oby.20224. 24493096 10.1002/oby.20224

[28] Sun Y, Liu B, Snetselaar LG, et al. Association of normal-weight central obesity with all-cause and cause-specific mortality among postmenopausal women. JAMA Netw Open. 2019;2(7):e197337. 10.1001/jamanetworkopen.2019.7337. 31339542 10.1001/jamanetworkopen.2019.7337 PMC6659146

[29] Papadakis GE, Hans D, Gonzalez Rodriguez E, et al. Menopausal hormone therapy is associated with reduced total and visceral adiposity: the osteolaus cohort. J Clin Endocrinol Metab. 2018;103(5):1948–57. 10.1210/jc.2017-02449. 29596606 10.1210/jc.2017-02449

[30] Chen Z, Bassford T, Green SB, et al. Postmenopausal hormone therapy and body composition–a substudy of the Estrogen plus progestin trial of the women’s health initiative. Am J Clin Nutr. 2005;82(3):651–6. 10.1093/ajcn.82.3.651. 16155280 10.1093/ajcn.82.3.651

[31] Espeland MA, Marcovina SM, Miller V, PEPI investigators, Postmenopausal estrogen/progestin interventions, et al. Effect of postmenopausal hormone therapy on lipoprotein(a) concentration. Circulation. 1998;97(10):979–86. 10.1161/01.cir.97.10.979. 10.1161/01.cir.97.10.979 9529266

[32] Casanova G, Bossardi Ramos R, Ziegelmann P, Spritzer PM. Effects of low-dose versus placebo or conventional-dose postmenopausal hormone therapy on variables related to cardiovascular risk: a systematic review and meta-analyses of randomized clinical trials. J Clin Endocrinol Metab. 2015;100(3):1028–37. 10.1210/jc.2014-3301. 25514104 10.1210/jc.2014-3301

[33] Bermingham KM, Linenberg I, Hall WL, et al. Menopause is associated with postprandial metabolism, metabolic health and lifestyle: the ZOE PREDICT study. EBioMedicine. 2022;85:104303. 10.1016/j.ebiom.2022.104303. 36270905 10.1016/j.ebiom.2022.104303 PMC9669773

[34] Heianza Y, Arase Y, Kodama S, et al. Effect of postmenopausal status and age at menopause on type 2 diabetes and prediabetes in Japanese individuals: Toranomon hospital health management center study 17 (TOPICS 17). Diabetes Care. 2013;36(12):4007–14. 10.2337/dc13-1048. 24170752 10.2337/dc13-1048 PMC3836104

[35] Mauvais-Jarvis F, Manson JE, Stevenson JC, Fonseca VA. Menopausal hormone therapy and type 2 diabetes prevention: evidence, mechanisms, and clinical implications. Endocr Rev. 2017;38(3):173–88. 10.1210/er.2016-1146. 28323934 10.1210/er.2016-1146 PMC5460681

[36] Pereira RI, Casey BA, Swibas TA, Erickson CB, Wolfe P, Van Pelt RE. Timing of estradiol treatment after menopause may determine benefit or harm to insulin action. J Clin Endocrinol Metab. 2015;100(12):4456–62. 10.1210/jc.2015-3084. 26425886 10.1210/jc.2015-3084 PMC4667161

[37] Speksnijder EM, Ten Noever Brauw GV, Malekzadeh A, Bisschop PH, Stenvers DJ, Siegelaar SE. Effect of postmenopausal hormone therapy on glucose regulation in women with type 1 or type 2 diabetes: a systematic review and meta-analysis. Diabetes Care. 2023;46(10):1866–75. 10.2337/dc23-0451. 10.2337/dc23-0451 37729504

[38] Matthews KA, Crawford SL, Chae CU, et al. Are changes in cardiovascular disease risk factors in midlife women due to chronological aging or to the menopausal transition? J Am Coll Cardiol. 2009;54(25):2366–73. 10.1016/j.jacc.2009.10.009. 20082925 10.1016/j.jacc.2009.10.009 PMC2856606

[39] de Aloysio D, Gambacciani M, Meschia M, The Icarus study group, et al. The effect of menopause on blood lipid and lipoprotein levels. Atherosclerosis. 1999;147(1):147–53. 10.1016/s0021-9150(99)00315-9. 10.1016/s0021-9150(99)00315-9 10525136

[40] Ambikairajah A, Walsh E, Cherbuin N. Lipid profile differences during menopause: a review with meta-analysis. Menopause N Y N. 2019;26(11):1327–33. 10.1097/GME.0000000000001403. 10.1097/GME.0000000000001403 31567869

[41] Fukami K, Koike K, Hirota K, Yoshikawa H, Miyake A. Perimenopausal changes in serum lipids and lipoproteins: a 7-year longitudinal study. Maturitas. 1995;22(3):193–7. 10.1016/0378-5122(95)00927-D. 8746876 10.1016/0378-5122(95)00927-d

[42] Peters HW, Westendorp IC, Hak AE, et al. Menopausal status and risk factors for cardiovascular disease. J Intern Med. 1999;246(6):521–8. 10.1046/j.1365-2796.1999.00547.x. 10620095 10.1046/j.1365-2796.1999.00547.x

[43] Torng PL, Su TC, Sung FC, et al. Effects of menopause and obesity on lipid profiles in middle-aged Taiwanese women: the Chin-Shan community cardiovascular cohort study. Atherosclerosis. 2000;153(2):413–21. 10.1016/s0021-9150(00)00423-8. 11164431 10.1016/s0021-9150(00)00423-8

[44] El Khoudary (سمر رياض الخضري). SR, Chen (陈曦润) X, Nasr (ألكسس نصر) A, HDL (High-Density Lipoprotein) Subclasses, Lipid Content, and Function Trajectories Across the Menopause Transition: SWAN-HDL Study. Arterioscler Thromb Vasc Biol. 2021;41(2):951–961.10.1161/ATVBAHA.120.315355. 10.1161/ATVBAHA.120.315355 PMC8105263 33267661

[45] Torosyan N, Visrodia P, Torbati T, Minissian MB, Shufelt CL. Dyslipidemia in midlife women: approach and considerations during the menopausal transition. Maturitas. 2022;166:14–20. 10.1016/j.maturitas.2022.08.001. 36027726 10.1016/j.maturitas.2022.08.001

[46] Lou Z, Huang Y, Lan Y, et al. Relationship between years since menopause and lipid variation in postmenopausal women: a cross-sectional study. Medicine. 2023;102(2):e32684. 10.1097/MD.0000000000032684. 36637918 10.1097/MD.0000000000032684 PMC9839288

[47] Derby CA, Crawford SL, Pasternak RC, Sowers M, Sternfeld B, Matthews KA. Lipid changes during the menopause transition in relation to age and weight: the study of women’s health across the Nation. Am J Epidemiol. 2009;169(11):1352–61. 10.1093/aje/kwp043. 19357323 10.1093/aje/kwp043 PMC2727246

[48] El Khoudary SR, Wang L, Brooks MM, Thurston RC, Derby CA, Matthews KA. Increase HDL-C level over the menopausal transition is associated with greater atherosclerotic progression. J Clin Lipidol. 2016;10(4):962–9. 10.1016/j.jacl.2016.04.008. 27578129 10.1016/j.jacl.2016.04.008 PMC5010007

[49] Rosenson RS, Brewer HB, Chapman MJ, et al. HDL measures, particle heterogeneity, proposed nomenclature, and relation to atherosclerotic cardiovascular events. Clin Chem. 2011;57(3):392–410. 10.1373/clinchem.2010.155333. 21266551 10.1373/clinchem.2010.155333

[50] Nie G, Yang X, Wang Y, et al. The effects of menopause hormone therapy on lipid profile in postmenopausal women: a systematic review and meta-analysis. Front Pharmacol. 2022;13:850815. 10.3389/fphar.2022.850815. 35496275 10.3389/fphar.2022.850815 PMC9039020

[51] Folsom AR, McGovern PG, Nabulsi AA, et al. Changes in plasma lipids and lipoproteins associated with starting or stopping postmenopausal hormone replacement therapy. Am Heart J. 1996;132(5):952–8. 10.1016/s0002-8703(96)90004-6. 8892766 10.1016/s0002-8703(96)90004-6

[52] Björnson E, Adiels M, Taskinen MR, et al. Lipoprotein(a) is markedly more atherogenic than LDL: an Apolipoprotein B-Based genetic analysis. J Am Coll Cardiol. 2024;83(3):385–95. 10.1016/j.jacc.2023.10.039. 38233012 10.1016/j.jacc.2023.10.039 PMC7616706

[53] Björnson E, Adiels M, Borén J, Packard CJ. Lipoprotein(a) is a highly atherogenic lipoprotein: pathophysiological basis and clinical implications. Curr Opin Cardiol. 2024;39(6):503–10. 10.1097/HCO.0000000000001170. 39360655 10.1097/HCO.0000000000001170

[54] Volgman AS, Koschinsky ML, Mehta A, Rosenson RS. Genetics and pathophysiological mechanisms of Lipoprotein(a)-associated cardiovascular risk. J Am Heart Assoc. 2024;13(12):e033654. 10.1161/JAHA.123.033654. 38879448 10.1161/JAHA.123.033654 PMC11255763

[55] Kronenberg F, Mora S, Stroes ESG, et al. Lipoprotein(a) in atherosclerotic cardiovascular disease and aortic stenosis: a European atherosclerosis society consensus statement. Eur Heart J. 2022;43(39):3925–46. 10.1093/eurheartj/ehac361. 36036785 10.1093/eurheartj/ehac361 PMC9639807

[56] Simony SB, Mortensen MB, Langsted A, Afzal S, Kamstrup PR, Nordestgaard BG. Sex differences of lipoprotein(a) levels and associated risk of morbidity and mortality by age: the Copenhagen general population study. Atherosclerosis. 2022;355:76–82. 10.1016/j.atherosclerosis.2022.06.1023. 35803767 10.1016/j.atherosclerosis.2022.06.1023

[57] Taskinen MR, Puolakka J, Pyörälä T, et al. Hormone replacement therapy lowers plasma Lp(a) concentrations. Comparison of cyclic transdermal and continuous estrogen-progestin regimens. Arterioscler Thromb Vasc Biol. 1996;16(10):1215–21. 10.1161/01.atv.16.10.1215. 8857916 10.1161/01.atv.16.10.1215

[58] Anagnostis P, Galanis P, Chatzistergiou V, et al. The effect of hormone replacement therapy and tibolone on lipoprotein (a) concentrations in postmenopausal women: a systematic review and meta-analysis. Maturitas. 2017;99:27–36. 10.1016/j.maturitas.2017.02.009. 28364865 10.1016/j.maturitas.2017.02.009

[59] Tsimikas S, Marcovina SM, Ancestry. Lipoprotein(a), and cardiovascular risk thresholds: JACC review topic of the week. J Am Coll Cardiol. 2022;80(9):934–46. 10.1016/j.jacc.2022.06.019. 36007992 10.1016/j.jacc.2022.06.019

[60] El Khoudary SR, Wildman RP, Matthews K, Thurston RC, Bromberger JT, Sutton-Tyrrell K. Progression rates of carotid intima-media thickness and adventitial diameter during the menopausal transition. Menopause. 2013;20(1):8–14. 10.1097/gme.0b013e3182611787. 22990755 10.1097/gme.0b013e3182611787 PMC3528819

[61] Samargandy S, Matthews KA, Brooks MM, et al. Arterial stiffness accelerates within 1 year of the final menstrual period: the SWAN heart study. Arterioscler Thromb Vasc Biol. 2020;40(4):1001–8. 10.1161/ATVBAHA.119.313622. 31969013 10.1161/ATVBAHA.119.313622 PMC7101253

[62] Detrano R, Guerci AD, Carr JJ, et al. Coronary calcium as a predictor of coronary events in four racial or ethnic groups. N Engl J Med. 2008;358(13):1336–45. 10.1056/NEJMoa072100. 18367736 10.1056/NEJMoa072100

[63] Budoff MJ, Young R, Burke G, et al. Ten-year association of coronary artery calcium with atherosclerotic cardiovascular disease (ASCVD) events: the multi-ethnic study of atherosclerosis (MESA). Eur Heart J. 2018;39(25):2401–8. 10.1093/eurheartj/ehy217. 29688297 10.1093/eurheartj/ehy217 PMC6030975

[64] Expert Panel on Cardiac Imaging, Ghoshhajra BB, Hedgire SS, et al. ACR appropriateness Criteria^®^ asymptomatic patient at risk for coronary artery disease: 2021 update. J Am Coll Radiol JACR. 2021;18(5S):S2–12. 10.1016/j.jacr.2021.01.003. 33958114 10.1016/j.jacr.2021.01.003

[65] Mehta A, Vasquez N, Ayers CR, et al. Independent association of lipoprotein(a) and coronary artery calcification with atherosclerotic cardiovascular risk. J Am Coll Cardiol. 2022;79(8):757–68. 10.1016/j.jacc.2021.11.058. 35210030 10.1016/j.jacc.2021.11.058 PMC10966924

[66] Fonseca MIH, de Almeida-Pititto B, Bittencourt MS, Bensenor IM, Lotufo PA, Ferreira SRG. Menopause per se is associated with coronary artery calcium score: results from the ELSA-Brasil. J Womens Health 2002. 2022;31(1):23–30. 10.1089/jwh.2021.0182. 10.1089/jwh.2021.0182 34520264

[67] Mackey RH, Kuller LH, Sutton-Tyrrell K, Evans RW, Holubkov R, Matthews KA. Lipoprotein subclasses and coronary artery calcium in postmenopausal women from the healthy women study. Am J Cardiol. 2002;90(8A):i71–6. 10.1016/s0002-9149(02)02636-x. 10.1016/s0002-9149(02)02636-x 12419483

[68] El Khoudary SR, Shields KJ, Janssen I, et al. Postmenopausal women with greater paracardial fat have more coronary artery calcification than premenopausal women: the study of women’s health across the Nation (SWAN) cardiovascular fat ancillary study. J Am Heart Assoc. 2017;6(2):e004545. 10.1161/JAHA.116.004545. 28137715 10.1161/JAHA.116.004545 PMC5523758

[69] Keshawarz A, Pyle L, Alman A, et al. Type 1 diabetes accelerates progression of coronary artery calcium over the menopausal transition: the CACTI study. Diabetes Care. 2019;42(12):2315–21. 10.2337/dc19-1126. 31558547 10.2337/dc19-1126 PMC6868458

[70] Rossouw JE, Anderson GL, Prentice RL, et al. Risks and benefits of Estrogen plus progestin in healthy postmenopausal women: principal results from the women’s health initiative randomized controlled trial. JAMA. 2002;288(3):321–33. 10.1001/jama.288.3.321. 12117397 10.1001/jama.288.3.321

[71] Manson JE, Allison MA, Rossouw JE, et al. Estrogen therapy and coronary-artery calcification. N Engl J Med. 2007;356(25):2591–602. 10.1056/NEJMoa071513. 17582069 10.1056/NEJMoa071513

[72] Manson JE, Chlebowski RT, Stefanick ML, et al. Menopausal hormone therapy and health outcomes during the intervention and extended poststopping phases of the women’s health initiative randomized trials. JAMA. 2013;310(13):1353–68. 10.1001/jama.2013.278040. 24084921 10.1001/jama.2013.278040 PMC3963523

[73] El Khoudary SR, Zhao Q, Venugopal V, et al. Effects of hormone therapy on heart fat and coronary artery calcification progression: secondary analysis from the KEEPS trial. J Am Heart Assoc. 2019;8(15):e012763. 10.1161/JAHA.119.012763. 31652073 10.1161/JAHA.119.012763 PMC6761637

[74] Simon A, Megnien JL, Chironi G. The value of carotid intima-media thickness for predicting cardiovascular risk. Arterioscler Thromb Vasc Biol. 2010;30(2):182–5. 10.1161/ATVBAHA.109.196980. 19948842 10.1161/ATVBAHA.109.196980

[75] Roman MJ, Naqvi TZ, Gardin JM, et al. Clinical application of noninvasive vascular ultrasound in cardiovascular risk stratification: a report from the American Society of Echocardiography and the Society of Vascular Medicine and Biology. J Am Soc Echocardiogr. 2006;19(8):943–54. 10.1016/j.echo.2006.04.020. 16880089 10.1016/j.echo.2006.04.020

[76] Harman SM, Black DM, Naftolin F, et al. Arterial imaging outcomes and cardiovascular risk factors in recently menopausal women: a randomized trial. Ann Intern Med. 2014;161(4):249–60. 10.7326/M14-0353. 25069991 10.7326/M14-0353

[77] Wang D, Jackson EA, Karvonen-Gutierrez CA, et al. Healthy lifestyle during the midlife is prospectively associated with less subclinical carotid atherosclerosis: the study of women’s health across the Nation. J Am Heart Assoc. 2018;7(23):e010405. 10.1161/JAHA.118.010405. 30482079 10.1161/JAHA.118.010405 PMC6405552

[78] Peila R, Xue X, LaMonte MJ, et al. Menopausal hormone therapy and change in physical activity in the women’s health initiative hormone therapy clinical trials. Menopause. 2023;30(9):898–905. 10.1097/GME.0000000000002231. 37527476 10.1097/GME.0000000000002231 PMC10527163

[79] Peltier MR, Flores JM, Smith PH, et al. Smoking across the menopausal transition in a 10-year longitudinal sample: the role of sex hormones and depressive symptoms. Nicotine Tob Res. 2020;22(6):872–7. 10.1093/ntr/ntz069. 31058288 10.1093/ntr/ntz069 PMC7249927

[80] Papadakis GE, Hans D, Gonzalez Rodriguez E, et al. The metabolic benefits of menopausal hormone therapy are not mediated by improved nutritional habits. OsteoLaus Cohort Nutrients. 2019;11(8):1930. 10.3390/nu11081930. 31426347 10.3390/nu11081930 PMC6722637

[81] Bushnell C, McCullough LD, Awad IA, et al. Guidelines for the prevention of stroke in women: a statement for healthcare professionals from the American Heart Association/American Stroke Association. Stroke. 2014;45(5):1545–88. 10.1161/01.str.0000442009.06663.48. 24503673 10.1161/01.str.0000442009.06663.48 PMC10152977

[82] Bushnell C, Kernan WN, Sharrief AZ, et al. 2024 guideline for the primary prevention of stroke: a guideline from the American Heart Association/American Stroke Association. Stroke. 2024;55(12):e344-424. 10.1161/STR.0000000000000475. 39429201 10.1161/STR.0000000000000475

[83] Gu H, Zhao X, Zhao X, Yang Y, Lv X. Risk of stroke in healthy postmenopausal women during and after hormone therapy: a meta-analysis. Menopause N Y N. 2014;21(11):1204–10. 10.1097/GME.0000000000000227. 10.1097/GME.0000000000000227 24686450

[84] Balasubramanian R, Demler O, Guasch-Ferré M, et al. Metabolomic effects of hormone therapy and associations with coronary heart disease among postmenopausal women. Circ Genom Precis Med. 2020;13(6):e002977. 10.1161/CIRCGEN.119.002977. 33141616 10.1161/CIRCGEN.119.002977 PMC8824616

[85] Shufelt CL, Manson JE. Menopausal hormone therapy and cardiovascular disease: the role of formulation, dose, and route of delivery. J Clin Endocrinol Metab. 2021;106(5):1245–54. 10.1210/clinem/dgab042. 33506261 10.1210/clinem/dgab042 PMC8063246

[86] Manson JE, Aragaki AK, Rossouw JE, et al. Menopausal hormone therapy and long-term all-cause and cause-specific mortality: the women’s health initiative randomized trials. JAMA. 2017;318(10):927–38. 10.1001/jama.2017.11217. 28898378 10.1001/jama.2017.11217 PMC5728370

[87] Walsh BW, Schiff I, Rosner B, Greenberg L, Ravnikar V, Sacks FM. Effects of postmenopausal estrogen replacement on the concentrations and metabolism of plasma lipoproteins. N Engl J Med. 1991;325(17):1196–204. 10.1056/NEJM199110243251702. 1922206 10.1056/NEJM199110243251702

[88] Grodstein F, Manson JE, Colditz GA, Willett WC, Speizer FE, Stampfer MJ. A prospective, observational study of postmenopausal hormone therapy and primary prevention of cardiovascular disease. Ann Intern Med. 2000;133(12):933–41. 10.7326/0003-4819-133-12-200012190-00008. 11119394 10.7326/0003-4819-133-12-200012190-00008

[89] Canonico M, Oger E, Plu-Bureau G, et al. Hormone therapy and venous thromboembolism among postmenopausal women: impact of the route of estrogen administration and progestogens: the ESTHER study. Circulation. 2007;115(7):840–5. 10.1161/CIRCULATIONAHA.106.642280. 17309934 10.1161/CIRCULATIONAHA.106.642280

[90] Smith NL, Blondon M, Wiggins KL, et al. Lower risk of cardiovascular events in postmenopausal women taking oral estradiol compared with oral conjugated equine estrogens. JAMA Intern Med. 2014;174(1):25–31. 10.1001/jamainternmed.2013.11074. 24081194 10.1001/jamainternmed.2013.11074 PMC4636198

[91] Shufelt CL, Merz CNB, Prentice RL, et al. Hormone therapy dose, formulation, route of delivery, and risk of cardiovascular events in women: findings from the women’s health initiative observational study. Menopause. 2014;21(3):260–6. 10.1097/GME.0b013e31829a64f9. 24045672 10.1097/GME.0b013e31829a64f9 PMC3872264

[92] Compounded Bioidentical Menopausal Hormone Therapy. ACOG clinical consensus 6. Obstet Gynecol. 2023;142(5):1266–73. 10.1097/AOG.0000000000005395. 37856860 10.1097/AOG.0000000000005395

[93] The NAMS 2020 GSM Position Statement Editorial Panel. The 2020 genitourinary syndrome of menopause position statement of the North American menopause society. Menopause N Y N. 2020;27(9):976–92. 10.1097/GME.0000000000001609. 10.1097/GME.0000000000001609 32852449

[94] Stefanick ML, Cochrane BB, Hsia J, Barad DH, Liu JH, Johnson SR. The women’s health initiative postmenopausal hormone trials: overview and baseline characteristics of participants. Ann Epidemiol. 2003;13(9 Suppl):S78–86. 10.1016/s1047-2797(03)00045-0. 14575940 10.1016/s1047-2797(03)00045-0

[95] Rossouw JE, Prentice RL, Manson JE, et al. Postmenopausal hormone therapy and risk of cardiovascular disease by age and years since menopause. JAMA. 2007;297(13):1465–77. 10.1001/jama.297.13.1465. 17405972 10.1001/jama.297.13.1465

[96] Nudy M, Aragaki AK, Jiang X, et al. Long-term changes to cardiovascular biomarkers after hormone therapy in the women’s health initiative hormone therapy clinical trials. Obstet Gynecol. 2025;145(4):357–67. 10.1097/AOG.0000000000005862. 40014858 10.1097/AOG.0000000000005862 PMC11972549

[97] Lobo RA. Where are we 10 years after the women’s health initiative?? J Clin Endocrinol Metab. 2013;98(5):1771–80. 10.1210/jc.2012-4070. 23493433 10.1210/jc.2012-4070

[98] Grady D, Applegate W, Bush T, Furberg C, Riggs B, Hulley SB. Heart and estrogen/progestin replacement study (HERS): design, methods, and baseline characteristics. Control Clin Trials. 1998;19(4):314–35. 10.1016/s0197-2456(98)00010-5. 9683309 10.1016/s0197-2456(98)00010-5

[99] Hulley S, Grady D, Bush T, et al. Randomized trial of Estrogen plus progestin for secondary prevention of coronary heart disease in postmenopausal women. Heart and estrogen/progestin replacement study (HERS) research group. JAMA. 1998;280(7):605–13. 10.1001/jama.280.7.605. 9718051 10.1001/jama.280.7.605

[100] Grady D, Herrington D, Bittner V, et al. Cardiovascular disease outcomes during 6.8 years of hormone therapy: heart and estrogen/progestin replacement study follow-up (HERS II). JAMA. 2002;288(1):49–57. 10.1001/jama.288.1.49. 12090862 10.1001/jama.288.1.49

[101] Schierbeck LL, Rejnmark L, Tofteng CL, et al. Effect of hormone replacement therapy on cardiovascular events in recently postmenopausal women: randomised trial. BMJ. 2012;345:e6409. 10.1136/bmj.e6409. 23048011 10.1136/bmj.e6409

[102] Hodis HN, Mack WJ, Henderson VW, et al. Vascular effects of early versus late postmenopausal treatment with estradiol. N Engl J Med. 2016;374(13):1221–31. 10.1056/NEJMoa1505241. 27028912 10.1056/NEJMoa1505241 PMC4921205

[103] ACOG Committee Opinion No. 565: Hormone therapy and heart disease. Obstet Gynecol. 2013;121(6):1407–1410. 10.1097/01.AOG.0000431053.33593.2d. 10.1097/01.AOG.0000431053.33593.2d 23812486

[104] O’Kelly AC, Michos ED, Shufelt CL, et al. Pregnancy and reproductive risk factors for cardiovascular disease in women. Circ Res. 2022;130(4):652–72. 10.1161/CIRCRESAHA.121.319895. 35175837 10.1161/CIRCRESAHA.121.319895 PMC8870397

[105] Cho L, Kaunitz AM, Faubion SS, et al. Rethinking menopausal hormone therapy: for whom, what, when, and how long? Circulation. 2023;147(7):597–610. 10.1161/CIRCULATIONAHA.122.061559. 36780393 10.1161/CIRCULATIONAHA.122.061559 PMC10708894

[106] Goff DC, Lloyd-Jones DM, Bennett G, et al. 2013 ACC/AHA guideline on the assessment of cardiovascular risk: a report of the American college of cardiology/American heart association task force on practice guidelines. Circulation. 2014;129(25 Suppl 2):S49-73. 10.1161/01.cir.0000437741.48606.98. 24222018 10.1161/01.cir.0000437741.48606.98

[107] Arnett DK, Blumenthal RS, Albert MA, et al. 2019 ACC/AHA guideline on the primary prevention of cardiovascular disease: a report of the American college of cardiology/american heart association task force on clinical practice guidelines. Circulation. 2019;140(11):e596-646. 10.1161/CIR.0000000000000678. 30879355 10.1161/CIR.0000000000000678 PMC7734661

[108] Wild RA, Hovey KM, Andrews C, et al. Cardiovascular disease (CVD) risk scores, age, or years since menopause to predict cardiovascular disease in the women’s health initiative. Menopause. 2021;28(6):610–8. 10.1097/GME.0000000000001753. 33950030 10.1097/GME.0000000000001753 PMC8141005

[109] Peterson LA, Freaney PM, Gulati M. Primary prevention and cardiovascular risk assessment in women. In: Shapiro MD, editor. Cardiovascular risk assessment in primary prevention. Springer International Publishing; 2022. pp. 177–97. 10.1007/978-3-030-98824-1_10.

[110] Bhatia HS, Becker RC, Leibundgut G, et al. Lipoprotein(a), platelet function and cardiovascular disease. Nat Rev Cardiol. 2024;21(5):299–311. 10.1038/s41569-023-00947-2. 37938756 10.1038/s41569-023-00947-2 PMC11216952

[111] An Update on Lp(a) and Aspirin in Primary Prevention - American College of Cardiology. https://www.acc.org/Latest-in-Cardiology/Articles/2024/07/17/14/02/An-Update-on-Lpa-and-Aspirin-in-Primary-Prevention.

[112] Bhatia HS, Trainor P, Carlisle S, et al. Aspirin and cardiovascular risk in individuals with elevated Lipoprotein(a): the multi-ethnic study of atherosclerosis. J Am Heart Assoc. 2024;13(3):e033562. 10.1161/JAHA.123.033562. 38293935 10.1161/JAHA.123.033562 PMC11056170

[113] Hirsch H, Manson JE. Menopausal symptom management in women with cardiovascular disease or vascular risk factors. Maturitas. 2022;161:1–6. 10.1016/j.maturitas.2022.01.016. 35688488 10.1016/j.maturitas.2022.01.016

[114] Hayes SN, Kim ESH, Saw J, et al. Spontaneous coronary artery dissection: current state of the science: A scientific statement from the American heart association. Circulation. 2018;137(19):e523–57. 10.1161/CIR.0000000000000564. 29472380 10.1161/CIR.0000000000000564 PMC5957087

[115] Olin JW, Gornik HL, Bacharach JM, et al. Fibromuscular dysplasia: state of the science and critical unanswered questions: a scientific statement from the American heart association. Circulation. 2014;129(9):1048–78. 10.1161/01.cir.0000442577.96802.8c. 24548843 10.1161/01.cir.0000442577.96802.8c

[116] Rikhi RR, Bhatia H. An Update on Lp(a) and Aspirin in Primary Prevention. American College of Cardiology. https://www.acc.org/Latest-in-Cardiology/Articles/2024/07/17/14/02/http%3a%2f%2fwww.acc.org%2fLatest-in-Cardiology%2fArticles%2f2024%2f07%2f17%2f14%2f02%2fAn-Update-on-Lpa-and-Aspirin-in-Primary-Prevention. Accessed 28 Jul 2025.

